# Transcriptomic profiling and discovery of key transcription factors involved in adventitious roots formation from root cuttings of mulberry

**DOI:** 10.1186/s12864-024-10593-8

**Published:** 2024-07-15

**Authors:** Hao Dou, Jiajia Sun, Tiantian Wang, Shuwen Bi, Xi Feng, Huijuan Sun, Jin’e Quan

**Affiliations:** College of Forest, Henan Agricultural University, Zhengzhou, 450002 China

**Keywords:** Mulberry, RNA-seq, DEGs, WGCNA, Transcription factors

## Abstract

ARs plays a crucial role in plant morphogenesis and development. The limited and inefficient rooting of scions poses a significant challenge to the efficiency and quality of clonal propagation of forest trees in silvicultural practices. Building on previous research conducted by our team, we found that applying IBA at a concentration of 1000 mg/L significantly enhanced mulberry rooting. This study aims to uncover the molecular mechanisms underlying this effect by analyzing RNA sequencing data from mulberry phloem before and after treatment with IBA over time intervals of 10, 20, 30, and 40 days. We identified 5226 DEGs, which were then classified into GO terms and KEGG pathways, showing significant enrichment in hormone signaling processes. Using WGCNA, we identified eight co-expression modules, two of which were significantly correlated with the IBA treatment. Additionally, 18 transcription factors that potentially facilitate ARs formation in mulberry were identified, and an exploratory analysis on the cis-regulatory elements associated with these transcription factors was conducted. The findings of this study provide a comprehensive understanding of the mechanisms of ARs in mulberry and offer theoretical support for the discovery and utilization of exceptional genetic resources within the species.

## Introduction

Adventitious roots (ARs), formed from non-root organs such as stems and leaves [1], enhance a plant’s ability to adapt to environmental changes and play a vital role in plant morphogenesis and development [2, 3]. The development of ARs in woody plants can be divided into three stages: dedifferentiation, induction, and differentiation [4]. During the dedifferentiation stage, parenchyma cells transform into embryogenic cells with a robust metabolism, which forms potential root primordia. In the induction stage, cells are stimulated to initiate cell division, forming clusters of meristematic cells that develop into visible root primordia. During the differentiation stage, cell differentiation occurs in a stratified manner. Multiple layers of root cap cells are produced at the apex of the root primordia, continuing to divide and differentiate into root tips. Meanwhile, posterior meristematic cells elongate to form vascular tissue, connecting with the vascular bundles in the original tissue. Ultimately, the root primordia protrude from the epidermal layer to generate ARs [5–7]. The recalcitrant nature of rooting and the scarcity of root formation are pivotal issues that impact the efficiency and quality of clonal propagation of forest trees in forestry operations [8].

Numerous studies have demonstrated that various phytohormones significantly influence the formation and development of ARs, with auxins, particularly indole-3-acetic acid (IAA) [9], having the most profound effect. Pei Dong and colleagues [10] have proposed that elevated IAA levels play a crucial role in the differentiation of root primordia during the initial phases of ARs induction. During this phase, IAA concentrations increase at the scion incision site, but these levels decrease once the root primordia are established [11]. Besides IAA, other phytohormones such as ethylene also play a role in AR formation, as demonstrated by its ability to stimulate root regeneration in species including chrysanthemum, petunia, and *Arabidopsis thaliana* [12–14]. The interaction between ethylene and IAA can synergistically enhance ARs genesis [15–17]. It has been shown that genes from the ethylene-responsive AP2/ERF transcription factor family are upregulated during the ARs induction period in poplar [18]. Conversely, cytokinins may antagonize auxin activity and inhibit AR development across various plant species [19–21], with higher IAA/cytokinin ratios being conducive to AR formation. Wang and collaborators [22] have found that abscisic acid can promote rooting in tetraploid acacia scions by counteracting the suppressive effects of high IAA concentrations. Additionally, Gutierrez and team [23] have shown that the auxin-responsive *Gretchen Hagen3* (*GH3*) gene family, specifically *GH3.3*, *GH3.5*, and *GH3.6*, are crucial for the fine-tuning of ARs initiation in Arabidopsis through the modulation of jasmonic acid homeostasis.

Advances in ARs research have moved from anatomical and physiological studies to the molecular level, largely driven by the development and integration of RNA sequencing (RNA-seq) technology. RNA-seq analysis has revealed that phytohormone signaling pathways are predominant in ARs development, as indicated in studies by Li Ke [24], who noted that exogenous indole-3-butyric acid (IBA) significantly induced hormone biosynthesis and responsive gene expression during ARs development in apple rootstocks. Similarly, Cheng Long’s RNA-seq studies [25] suggested that aluminum exposure might facilitate the regeneration and development of ARs in tea plants through a complex transcriptional regulatory network involving various plant hormones and associated genes. However, research on the molecular mechanisms of ARs formation has primarily focused on model plants like Arabidopsis and rice, with limited studies on mulberry.

Mulberry has been cultivated in China for a long time, and with advancements in research methodologies, our understanding of its properties has deepened. Mulberry leaves and fruits are known for their high nutritional value and health benefits [26–28]. Although mulberry cuttings traditionally exhibit low survival rates, advancements in cutting techniques have significantly improved their viability [29, 30]. However, the generation and quality of roots remain major challenges. Quickly forming mature roots in cutting seedlings is currently an urgent issue to address [31].

According to prior research conducted by our group, the application of IBA at a concentration of 1000 mg/L was found to be most effective for mulberry rooting [32]. To explore the molecular mechanisms, RNA-seq was performed at intervals before and after the 1000 mg/L IBA treatment, leading to the identification of differentially expressed genes (DEGs) categorized into Gene Ontology (GO) terms and Kyoto Encyclopedia of Genes and Genomes (KEGG) pathways. Subsequently, a transcription factor (TF) gene regulatory network was constructed from these DEGs using Weighted Gene Correlation Network Analysis (WGCNA). The enrichment of DEGs in GO terms and KEGG pathways, along with the construction of the TF gene regulatory network based on WGCNA, aims to provide both practical and theoretical insights for the propagation and rooting mechanisms of mulberry cuttings, potentially benefiting the cultivation practices of other woody plant species.

## Materials and methods

### Experimental site, plant materials and experimental design

The experiment was carried out at the third living area of Henan Agricultural University located in Zhengzhou City, Henan Province. The site is situated at geographical coordinates of 113.22°E longitude, 34.28°N latitude, with an elevation of 98 m above sea level. The cuttings were sourced from a greenhouse specifically designed for propagation, which was equipped with comprehensive full-spectrum lighting and an automatic misting system. The greenhouse contained cutting pools divided into five sections, each approximately 11 m by 6 m, with each sub-pool measuring 6 m by 2 m and having a depth of 0.4 m.

**Fig. 1:**
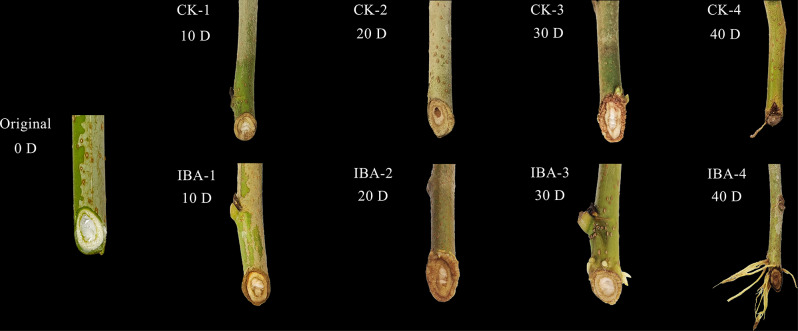
Changes in root morphology of mulberry across four periods

Cuttings were taken from semi-lignified branches of the mulberry cultivar “Qiangsang No. 1,” developed by the Silkworm Research Institute of the Zhejiang Academy of Agricultural Sciences. These were processed into uniformly sized stakes, and the basal ends were treated with either a 1000 mg/L IBA solution for the treatment group or water for the control group (CK) for 30 s. Each treatment was replicated three times, using 80 cuttings per replicate, all cuttings were guaranteed to come from a uniform clone Soaked all the branches in carbendazim for 1 ~ 2 min, put the cuttings in a cool and ventilated place, dry the liquid in the shade. The prepared cuttings were then inserted into a sterilized growth medium according to established protocols detailed in our previous publication [32]. The developmental stages of the softwood cuttings’ rooting process were documented, as illustrated in Fig. 1. This documentation included stages of callus formation from 0 to 10 days post-planting, induction of root primordia from 10 to 20 days, emergence and formation of ARs from 20 to 30 days, and elongation and maturation of ARs beyond 40 days. For the study of ARs development, cortical tissue samples approximately 1 cm above the base of the cuttings were harvested at 10 (CK-1, IBA-1), 20 (CK-2, IBA-2), 30 (CK-3, IBA-3), and 40 days (CK-4, IBA-4) post-planting for both the control and treatment groups. The collected samples were immediately immersed in liquid nitrogen and subsequently stored in a -80 °C freezer. Twenty specimens were randomly selected from each time point and treatment for transcriptome analysis.

### RNA sequencing

For transcriptome sequencing (RNA-seq), three biological replicates were collected from both the control and IBA-treated groups at each time point. In total, 24 RNA-seq libraries (two treatments × four time points × three biological replicates) were generated. Total RNA was isolated using TRIzol reagent, and the libraries were constructed and sequenced on an Illumina HiSeq™ 2500 platform at BMK Company, Beijing, China. Raw sequence reads, comprising 150 bp paired-end reads, were filtered and aligned as previously described by Ahmad and colleagues [33].

### Sequence alignment to the *Morus notabilis* genome and RNA-sequencing data analysis

Following sequencing, high-quality reads were obtained by removing adapter sequences, low-quality reads, and ambiguous nucleotides (N). Concurrently, during the trimming and filtering process, descriptive statistics for the resultant high-quality data were calculated, including Q20, Q30 scores, GC content, and the level of sequence duplication. These high-quality reads were then used for further analysis. They were aligned to the *Morus notabilis* reference genome available at the *Morus notabilis* reference genome (https://morus.biodb.org/browse) using HISAT2 software [34]. DEGs were identified by calculating the log2 fold-change (FC) of gene expression at different treatment stages. DEGs were selected based on |log2(FC)| ≥ 2 and a statistical significance threshold of *P* ≤ 0.05. The DESeq tool in R was employed to detect DEGs using the criteria of |log2 ratio| ≥ 1 and an adjusted P-value (false discovery rate, FDR) ≤ 0.05 [35, 36].

The KEGG (http://www.kegg.jp, accessed on 13 October 2023) and GO (http://geneontology.org, accessed on 28 October 2023) databases were utilized to perform enrichment analyses of transcripts and DEGs per sample. KEGG facilitates the prediction of protein interaction networks and their functions in various cellular processes. GO enrichment analysis was applied to categorize the primary biological functions of the DEGs in terms of molecular functions, cellular components, and biological processes. The hypergeometric test was used to identify significantly enriched pathways and GO terms among the DEGs compared to the genomic background. The resultant P-values were adjusted to control the FDR, with an FDR ≤ 0.05 considered significant. The DESeq R package was employed to apply the hypergeometric test for enrichment analysis.

### Weighted gene co-expression network analysis

WGCNA was conducted based on the expression correlation patterns among DEGs. The DEGs were analyzed using the log2-transformed FPKM values plus one as input, and the soft thresholding power was determined by the scale-free network criterion [37]. The lowest power value at which the scale independence reached a plateau (or exceeded 0.8) was chosen for downstream analysis, and the changes in gene connectivity at various power values were also examined [38–40]. Genes were clustered into modules using dynamic tree cutting. A gene clustering dendrogram was constructed based on gene expression correlations, and gene modules were defined according to the clustering dendrogram. Modules with similar expression profiles were then merged based on the similarity of their module eigengenes, with a minimum of 50 genes per module and a merging threshold of 0.8. Modules were identified as significant through module eigengene analysis, and relevant modules were selected for more detailed investigation.

### Validation of DEGs by RT‒qPCR

Real-time quantitative PCR (RT‒qPCR) was used to validate the transcriptome data, and 10 DEGs were randomly selected for RT‒qPCR. The Actin gene was used as an internal reference gene [41]. Specific primers: 5’-F: GGTTCTCCTGACTGAGGCAC-3’, R: 5’-F: AGTCAAGAACGATACCAGTCGT-3’. Primers were designed according to the sequences of each gene (Table 1), and the differential gene expression levels were detected on the Bio-Rad Real-Time Fluorescence Quantitative PCR Instrument. The RT-qPCR system consisted of 2 × RealStar Green Fast Mixture 10 µL(Genstar, Beijing, China), template cDNA 1 µL, forward/reverse primers 0.5 µL (10 µmol/L) each, ddH2O 8 µL PCR program: 94 ℃ predenaturation 2 min; 94 ℃ denaturation 15 s, 60 ℃ annealing30 s, cycling 40 times. The relative gene expression was calculated by the 2^ΔΔCt^ method [42].

**Table 1:** Primers for quantitative PCR analysis

Gene		sequence(5’-3’)	Product size
Actin	F	GGTTCTCCTGACTGAGGCAC	158 bp
	R	AGTCAAGAACGATACCAGTCGT	
gene13294	F	GTGTCGTGACGGCTTATTATATGTG	143 bp
	R	TCGTCCACCAGTCCCATTCT	
gene2183	F	TAACATTCGATCCCGACCGC	135 bp
	R	AGTCTCGGTCGAATCCTGG	
gene2778	F	CACCCCAACACAAGGAAACG	165 bp
	R	ACAGGTTCGTACAAGGGACG	
gene8053	F	AGTGATCTCAAAAACAGTTCGGTG	134 bp
	R	GAGAGGTCGTGGATCGTCAC	
gene12859	F	TGCCCGACATTCCTCAACTT	147 bp
	R	CCACTCTCTCCACTTTCTGTTGT	
gene14916	F	TCCATCCGACCGAGGAAGAG	133 bp
	R	ATCTTCGCCTTCCCAGGCA	
gene16958	F	GGTGCCAATGTCCAGGTGTG	115 bp
	R	TCTCCACAGCCTTCTCAGGG	
gene18740	F	CGAGGGAATCTGTACGAGCA	132 bp
	R	CGGTACTCCTCCACCATCCT	
gene23141	F	TGCCTCAACAAGCCGAGATT	97 bp
	R	ACCTTCTTCTGCTGATTTTTCCTCT	
gene23176	F	GATGATGGGCTTCTCAGGCA	129 bp
	R	AAGAAGCCAAAAGCCAGAGC	

## Results and analysis

### Sequencing data quality control and comparison of reference genomes

The 24 RNA-seq libraries that were generated underwent analysis, and the short sequences produced through sequencing constituted the raw data. Given that RNA extraction, library preparation, and sequencing can introduce redundant or low-quality data, clean data were acquired by filtering the raw data to remove duplicated reads, reads containing adapters, reads with a high proportion of N, and low-quality reads. This process involved quality assessment and control. From the 24 samples, a total of 150.20 Gb of valid data was obtained, averaging 5.02 Gb per sample, with an individual sample data size of around 7.14 Gb on average. These data were saved in the FastQ file format, facilitating the smooth progression of subsequent bioinformatics analyses. The data output statistics for each sample are presented in the accompanying table (Table 2). Post sequencing quality control, a total of 150,197,323,354 clean data points were collected. The GC content for each sample ranged between 45.33% and 46.67%, and the percentage of Q30 bases in each sample was no less than 89.45%. These metrics indicated that the sequencing results of the “Qiangsang No. 1” spikelet samples were highly reliable, of superior quality, and provided a robust data set suitable for further assembly and analysis. The quality-controlled clean reads were then mapped to the *Morus notabilis* genome, with the alignment efficiency of each sample’s reads to the reference genome ranging from 63.71 to 75.52% (Table 3).

**Table 2:** Statistical tables of sequencing data

Samples	Clean reads	Clean bases	GC Content	%≥Q 30
CK-A-1	20,391,884	6,098,641,606	0.4533	0.8962
CK-A-2	19,624,220	5,868,650,874	0.458	0.905
CK-A-3	20,620,542	6,167,568,604	0.4556	0.9067
CK-B-1	20,432,673	6,111,394,980	0.4594	0.9031
CK-B-2	20,394,654	6,097,994,444	0.4626	0.9078
CK-B-3	22,096,179	6,608,679,454	0.4613	0.9067
CK-C-1	19,179,456	5,736,780,012	0.4544	0.9013
CK-C-2	20,155,109	6,028,694,862	0.4556	0.9078
CK-C-3	20,693,054	6,189,994,430	0.4612	0.9065
CK-D-1	20,509,243	6,133,065,196	0.4563	0.9066
CK-D-2	20,568,878	6,151,347,440	0.4603	0.909
CK-D-3	19,609,641	5,864,808,108	0.4582	0.9051
IBA-1-1	23,188,344	6,935,112,014	0.465	0.9129
IBA-1-2	24,789,794	7,417,506,198	0.4608	0.9125
IBA-1-3	21,873,062	6,544,606,038	0.4667	0.9179
IBA-2-1	20,377,357	6,093,960,084	0.46	0.9058
IBA-2-2	21,459,646	6,417,837,058	0.4564	0.8995
IBA-2-3	21,262,767	6,358,677,938	0.4613	0.9133
IBA-3-1	20,411,779	6,106,187,162	0.458	0.896
IBA-3-2	20,710,008	6,195,531,550	0.4591	0.9164
IBA-3-3	20,574,023	6,154,672,398	0.4629	0.9057
IBA-4-1	20,552,516	6,147,395,690	0.4609	0.9099
IBA-4-2	21,706,184	6,493,013,408	0.465	0.9025
IBA-4-3	20,976,416	6,275,203,806	0.4584	0.9125

**Table 3:** Statistics of sequence comparison results of sample sequencing data with selected reference genomes

Samples	Total Reads	Mapped Reads	Uniq Mapped Reads	Multiple Map Reads	Reads Map to ‘+’	Reads Map to ‘-’
CK-A-1	40,783,768	29,038,210 (71.20%)	27,935,655 (68.50%)	1,102,555 (2.70%)	15,242,917 (37.37%)	15,178,636 (37.22%)
CK-A-2	39,248,440	28,340,694 (72.21%)	27,211,253 (69.33%)	1,129,441 (2.88%)	14,917,343 (38.01%)	14,804,196 (37.72%)
CK-A-3	41,241,084	29,549,355 (71.65%)	28,304,208 (68.63%)	1,245,147 (3.02%)	15,575,254 (37.77%)	15,466,766 (37.50%)
CK-B-1	40,865,346	28,916,083 (70.76%)	27,748,585 (67.90%)	1,167,498 (2.86%)	15,143,295 (37.06%)	15,140,565 (37.05%)
CK-B-2	40,789,308	29,291,250 (71.81%)	28,025,563 (68.71%)	1,265,687 (3.10%)	15,453,014 (37.88%)	15,402,145 (37.76%)
CK-B-3	44,192,358	31,356,973 (70.96%)	30,040,893 (67.98%)	1,316,080 (2.98%)	16,471,789 (37.27%)	16,481,663 (37.30%)
CK-C-1	38,358,912	28,296,096 (73.77%)	27,334,420 (71.26%)	961,676 (2.51%)	14,688,816 (38.29%)	14,721,527 (38.38%)
CK-C-2	40,310,218	29,625,404 (73.49%)	28,594,270 (70.94%)	1,031,134 (2.56%)	15,416,935 (38.25%)	15,404,123 (38.21%)
CK-C-3	41,386,108	30,557,310 (73.83%)	29,407,960 (71.06%)	1,149,350 (2.78%)	15,958,772 (38.56%)	15,947,953 (38.53%)
CK-D-1	41,018,486	30,186,837 (73.59%)	29,053,487 (70.83%)	1,133,350 (2.76%)	15,808,779 (38.54%)	15,738,863 (38.37%)
CK-D-2	41,137,756	30,415,840 (73.94%)	29,181,482 (70.94%)	1,234,358 (3.00%)	16,004,331 (38.90%)	15,914,966 (38.69%)
CK-D-3	39,219,282	29,306,286 (74.72%)	28,300,795 (72.16%)	1,005,491 (2.56%)	15,267,397 (38.93%)	15,277,143 (38.95%)
IBA-1-1	46,376,688	34,460,957 (74.31%)	32,934,670 (71.02%)	1,526,287 (3.29%)	18,260,677 (39.37%)	18,220,685 (39.29%)
IBA-1-2	49,579,588	37,098,190 (74.83%)	35,536,883 (71.68%)	1,561,307 (3.15%)	19,568,506 (39.47%)	19,531,961 (39.40%)
IBA-1-3	43,746,124	33,036,429 (75.52%)	31,455,507 (71.90%)	1,580,922 (3.61%)	17,644,489 (40.33%)	17,480,041 (39.96%)
IBA-2-1	40,754,714	29,626,355 (72.69%)	28,468,385 (69.85%)	1,157,970 (2.84%)	15,484,194 (37.99%)	15,507,595 (38.05%)
IBA-2-2	42,919,292	30,798,051 (71.76%)	29,655,582 (69.10%)	1,142,469 (2.66%)	16,052,403 (37.40%)	16,081,549 (37.47%)
IBA-2-3	42,525,534	30,701,678 (72.20%)	29,441,814 (69.23%)	1,259,864 (2.96%)	16,106,423 (37.87%)	16,078,257 (37.81%)
IBA-3-1	40,823,558	29,585,694 (72.47%)	28,500,721 (69.81%)	1,084,973 (2.66%)	15,415,435 (37.76%)	15,444,505 (37.83%)
IBA-3-2	41,420,016	30,819,202 (74.41%)	29,462,310 (71.13%)	1,356,892 (3.28%)	16,306,435 (39.37%)	16,217,123 (39.15%)
IBA-3-3	41,148,046	30,176,009 (73.34%)	28,922,519 (70.29%)	1,253,490 (3.05%)	15,901,561 (38.64%)	15,759,097 (38.30%)
IBA-4-1	41,105,032	30,497,721 (74.19%)	29,421,584 (71.58%)	1,076,137 (2.62%)	15,848,232 (38.56%)	15,922,032 (38.73%)
IBA-4-2	43,412,368	31,318,231 (72.14%)	30,119,482 (69.38%)	1,198,749 (2.76%)	16,310,498 (37.57%)	16,412,243 (37.81%)
IBA-4-3	41,952,832	31,543,013 (75.19%)	30,370,153 (72.39%)	1,172,860 (2.80%)	16,485,067 (39.29%)	16,501,104 (39.33%)

### Repeat relevance assessment

**Fig. 2:**
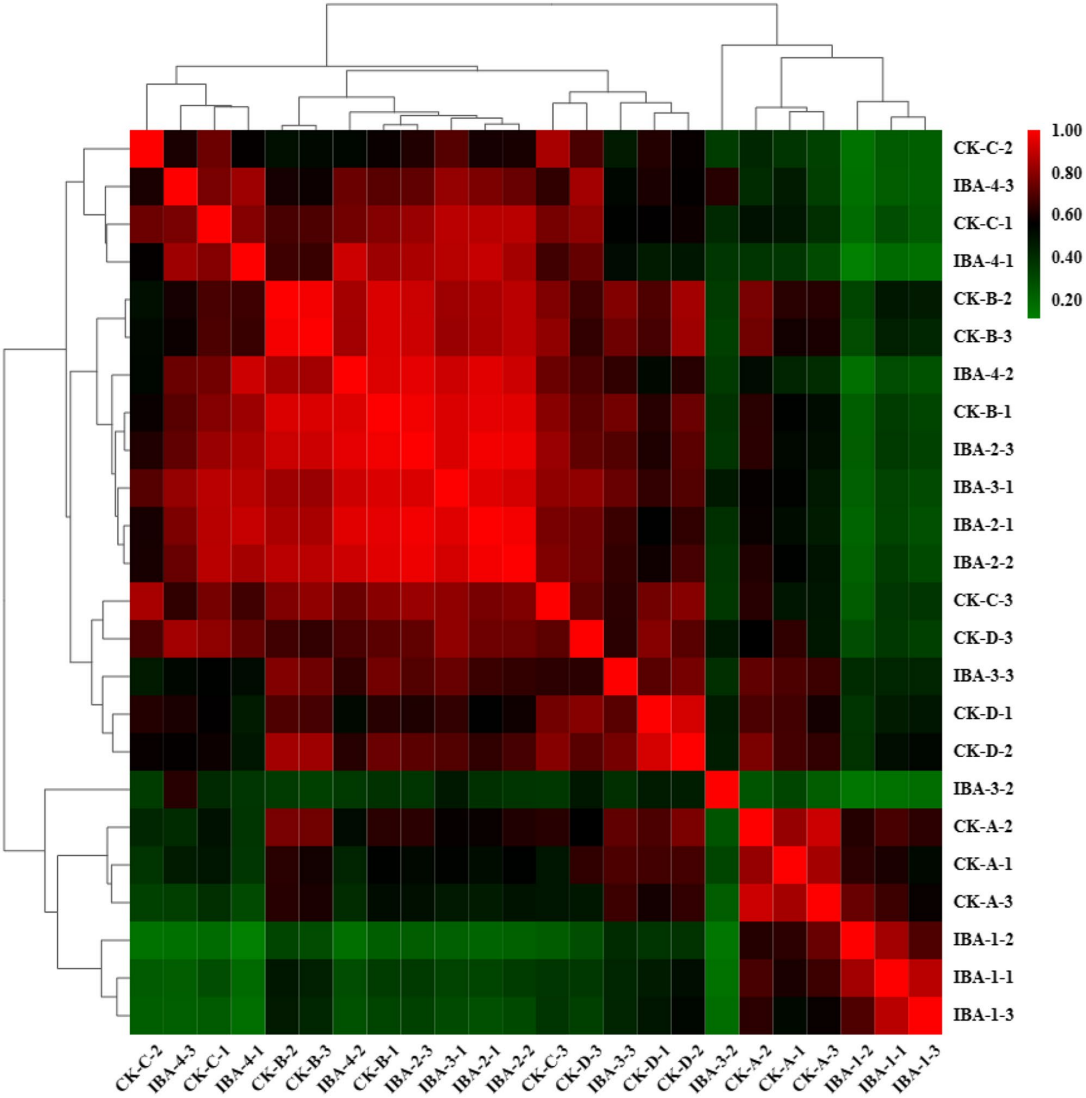
Expression correlation heatmap of pairwise samples

To identify differentially expressed genes of genuine interest, it is necessary to account for and mitigate the impact of this biological variability. In this study, the correlation between gene expression levels across samples serves as a critical metric for evaluating the reproducibility of the biological experiments, confirming the validity of the identified differentially expressed genes, and aiding in the identification of outlier samples. We employed Pearson’s correlation coefficient (r) as the measure of correlation between biological replicates [43], with an r2 value approaching 1 denoting a strong correlation between two replicate samples. The heatmap depicting the sample correlations in this study is presented in Fig. 2. The results indicated that most replicates clustered together, suggesting that the biological replicates were generally well-established. The highest correlation was observed between the treatment groups across different time points, while a lower correlation was apparent within the CK, which also suggests that the gene expression in the samples underwent significant changes post-treatment, consistent with the expected pattern of the experiment.

### Analysis of EDGs in treatment and expression groups

**Fig. 3:**
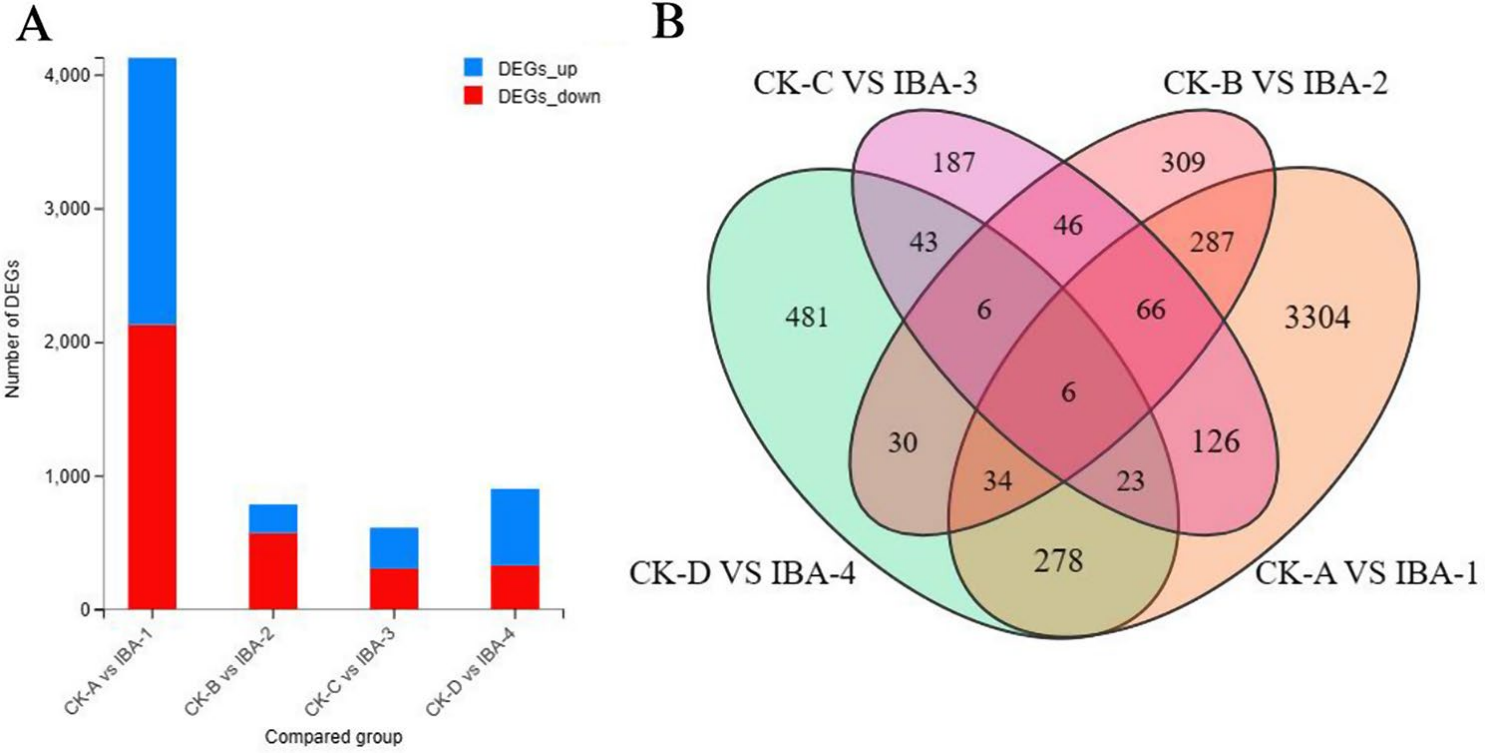
Analyses of differentially expressed genes (DEGs)

Before and after treatment with 1000 mg/L^− 1^ IBA, a total of 5,226 DEGs were identified across the four periods. Specifically, the comparison between CK-A and IBA-1 revealed a total of 4,124 DEGs, with 1,995 upregulated and 2,129 downregulated; CK-B vs. IBA-2 yielded a total of 784 DEGs, with 213 upregulated and 571 downregulated; CK-C vs. IBA-3 resulted in 503 DEGs, with 305 upregulated and 198 downregulated; and CK-D vs. IBA-4 achieved a total of 5226 DEGs. 305 DEGs and 198 downregulated DEGs were obtained; CK-D vs. IBA-4 identified 901 DEGs, of which 572 were upregulated and 329 were downregulated. The comparisons CK-A vs. IBA-1 and CK-B vs. IBA-2 shared 393 DEGs, CK-B vs. IBA-2 and CK-C vs. IBA-3 shared 124 DEGs, CK-C vs. IBA-3 and CK-D vs. IBA-4 shared 78 DEGs, and CK-A vs. IBA-1 and CK-D vs. IBA-4 had 341 DEGs in common (Fig. 3). There were six genes common to all four comparison groups, suggesting that these DEGs continued to exhibit significant changes throughout the course of the treatment with IBA.

**Fig. 4:**
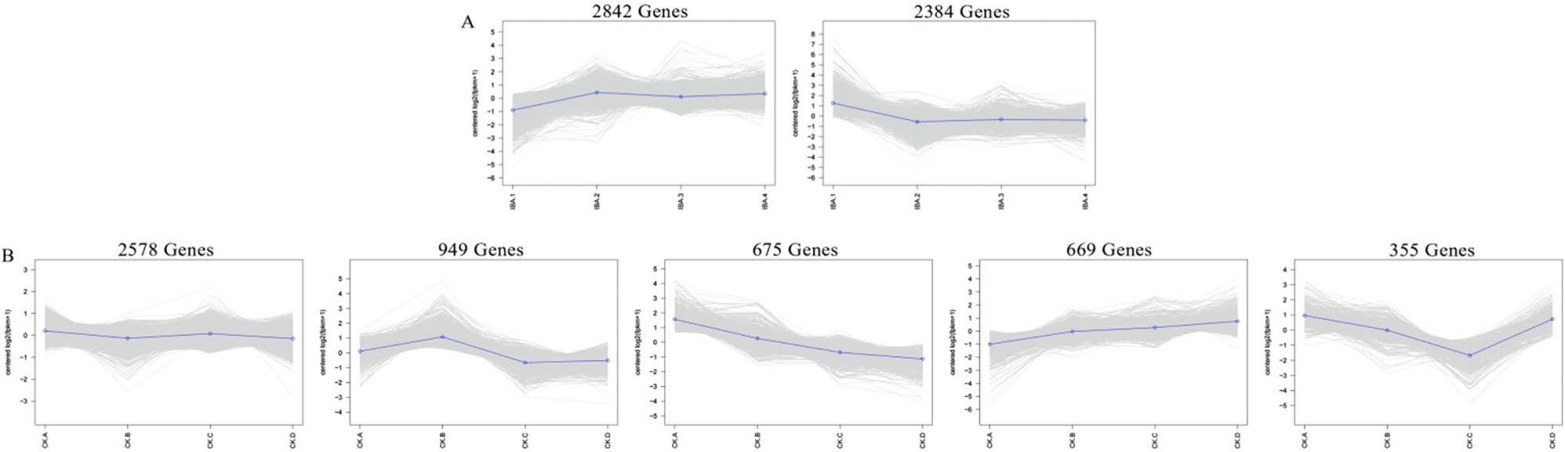
Trend analysis of gene coexpression of all DEGs over eight periods in two treatments. All DEGs were categorized into two expression trends (**A**, **B**)

In parallel, this study utilized STEM software to normalize the expression data based on log2(fpkm + 1) using the expression counts. This normalization was then used to analyze the expression trends of the 4,124 DEGs in both the CK group and the treatment group, Fig. 4 shows two different trends The analysis revealed two distinct expression patterns with significant differences in expression levels among these DEGs in the treatment group, while five distinct expression patterns with significant differences were observed in the CK group. The trends in expression of these DEGs in the IBA treatment group and the CK group were markedly different.

### GO and KEGG analysis of DEGs

**Fig. 5:**
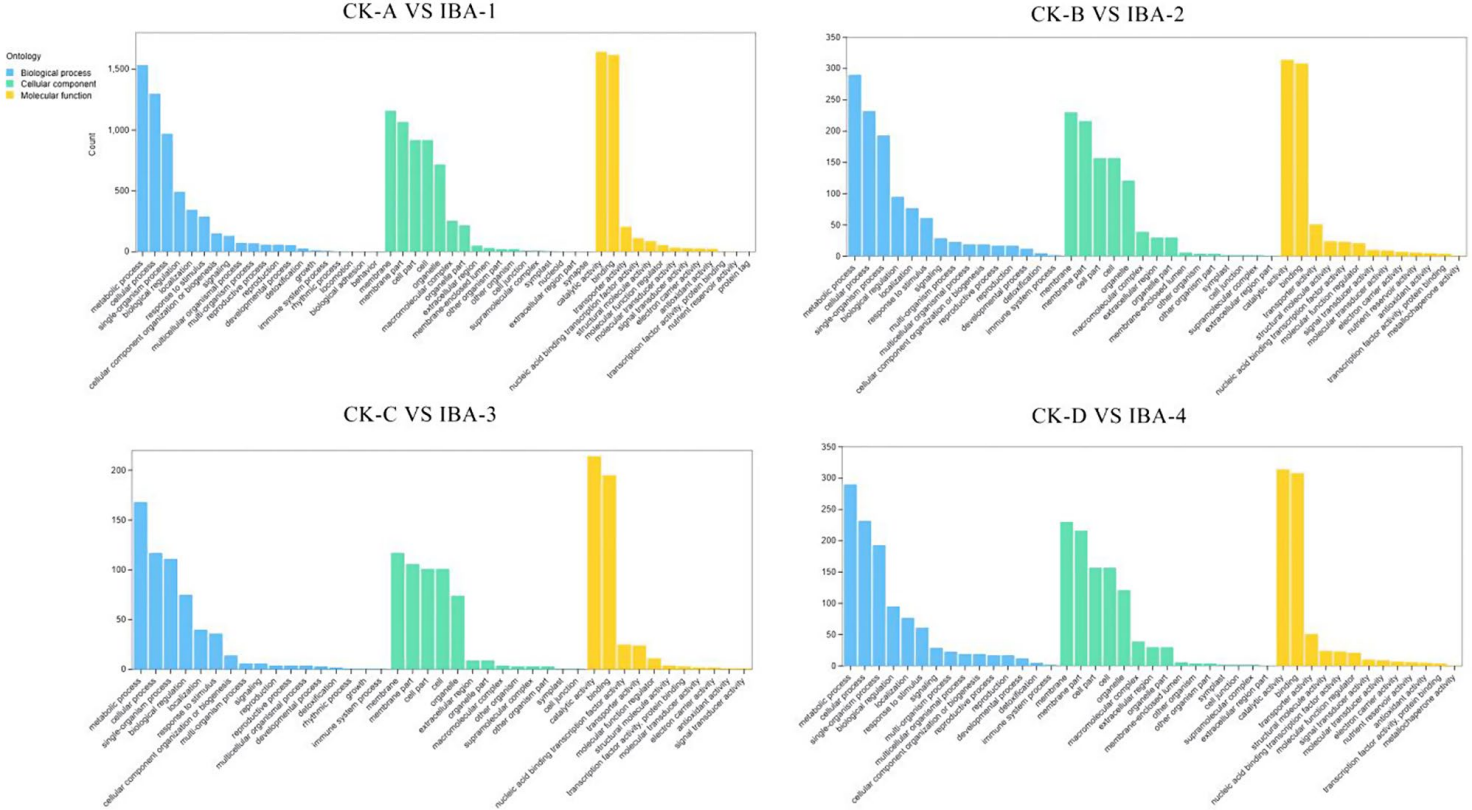
GO enrichment analysis of DEGs from four time periods

To elucidate the molecular mechanisms in mulberry following IBA treatment, GO and KEGG analyses were conducted on differentially expressed genes (DEGs) from four temporal stages. GO enrichment analysis of the top 20 categories (Fig. 5) categorizes DEGs into three domains: biological process, cellular component, and molecular function. Within the biological process domain, metabolic process (GO: 0008152), cellular process (GO: 0009987), and single-organism process (GO: 0044699) were significantly enriched; for the cellular component domain, components such as membrane (GO: 0016020), cell (GO: 0005623), and cell part (GO: 0044464) were significantly enriched; and within the molecular function domain, binding (GO: 0005488), catalytic activity (GO: 0003824), and transporter activity (GO: 0005215) were significantly enriched. The results of the GO enrichment for the four stages were highly similar, indicating that extensive cellular metabolic activity occurred throughout all stages.

**Fig. 6:**
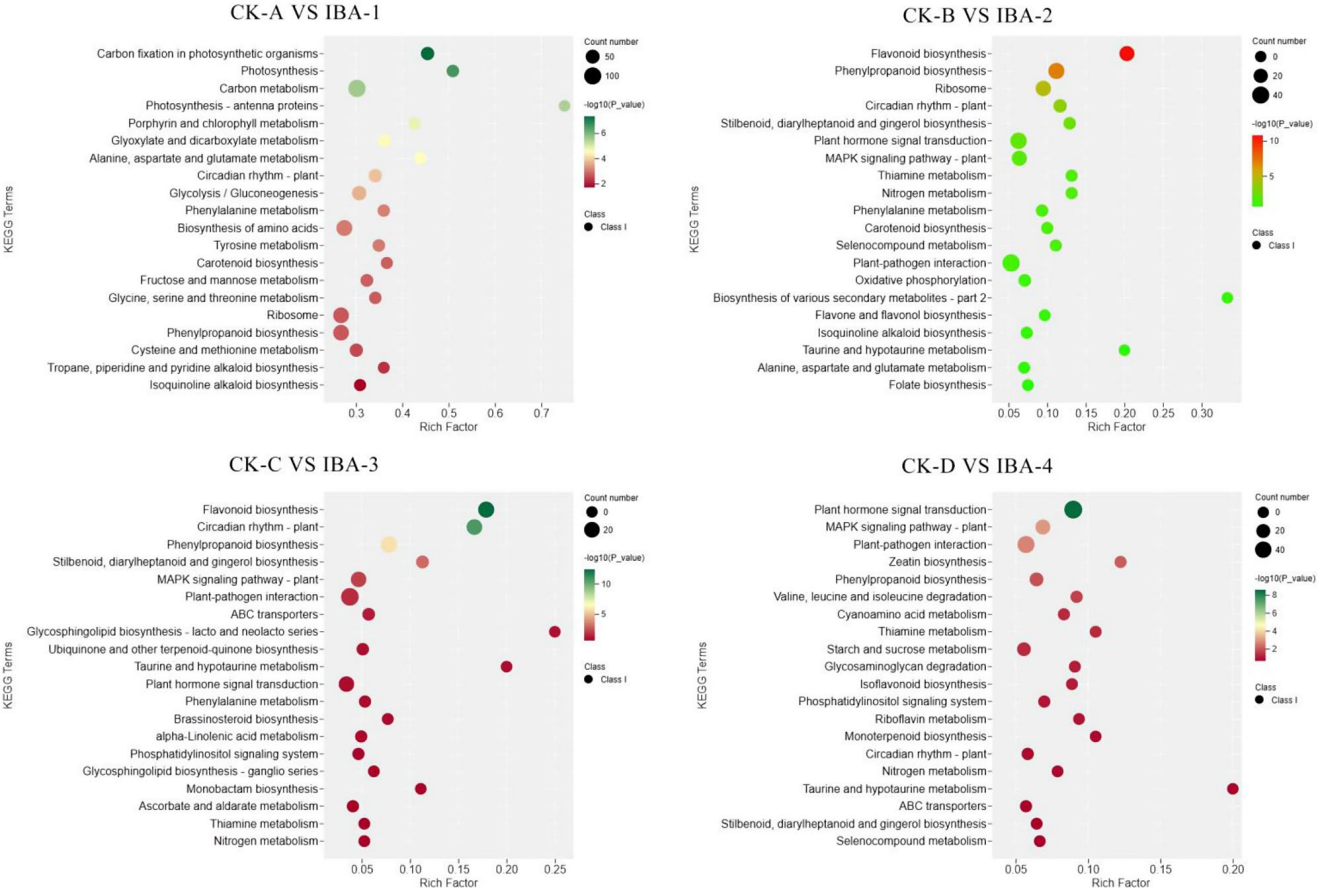
KEGG enrichment analysis of DEGs from four time periods

Diverging from the GO annotations, the KEGG pathway enrichment results varied across the four stages (Fig. 6). In the comparison between CK-A and IBA-1, the pathways of photosynthesis - antenna proteins (ko00196), taurine and hypotaurine metabolism (ko00430), and caffeine metabolism (ko00232) were significantly enriched. In the comparison between CK-B and IBA-2, pathways including biosynthesis of various secondary metabolites (ko00998), flavonoid biosynthesis (ko00941), and taurine and hypotaurine metabolism (ko00430) were notably enriched. For CK-C vs. IBA-3, pathways such as glycosphingolipid biosynthesis - lacto and neolacto series (ko00601), taurine and hypotaurine metabolism (ko00430), and flavonoid biosynthesis (ko00941) were significantly enriched. Finally, in the comparison between CK-D and IBA-4, pathways like taurine and hypotaurine metabolism (ko00430), zeatin biosynthesis (ko00908), and synthesis and degradation of ketone bodies (ko00072) were significantly enriched. Collectively, these results suggest that most DEGs were associated with secondary metabolite synthesis pathways under IBA treatment, indicating a strong engagement in organic matter metabolism. Remarkably, after analyzing the number of genes enriched in each pathway, the pathway with the highest number of enriched genes was plant hormone signal transduction (Table 4), further suggesting that the phytohormone pathway is significantly activated following IBA treatment. This observation aligns with the findings from the aforementioned ARs production study and underscores plant hormone signal transduction as a pivotal focus for further research.

**Table 4:** Statistics of the number of DEGs enriched in the KEGG pathway

Different rooting stages	Pathway DES	Pathway ID	genes numbers
CK-A VS IBA-1	Plant hormone signal transduction	ko04075	164
	Plant‒pathogen interaction	ko04626	127
	Carbon metabolism	ko01200	103
	MAPK signaling pathway - plant	ko04016	88
	Biosynthesis of amino acids	ko01230	80
	Phenylpropanoid biosynthesis	ko00940	79
	Ribosome	ko03010	79
	Starch and sucrose metabolism	ko00500	66
	Glycolysis/Gluconeogenesis	ko00010	58
	Protein processing in endoplasmic reticulum	ko04141	58
CK-B VS IBA-2	Plant hormone signal transduction	ko04075	41
	Plant‒pathogen interaction	ko04626	35
	Phenylpropanoid biosynthesis	ko00940	33
	Ribosome	ko03010	28
	MAPK signaling pathway - plant	ko04016	26
	Flavonoid biosynthesis	ko00941	25
	Circadian rhythm - plant	ko04712	14
	Pentose and glucuronate interconversions	ko00040	10
	Starch and sucrose metabolism	ko00500	10
	Oxidative phosphorylation	ko00190	9
CK-C VS IBA-3	Plant‒pathogen interaction	ko04626	29
	Phenylpropanoid biosynthesis	ko00940	23
	Flavonoid biosynthesis	ko00941	22
	Circadian rhythm - plant	ko04712	20
	MAPK signaling pathway - plant	ko04016	19
	Plant hormone signal transduction	ko04075	19
	Starch and sucrose metabolism	ko00500	9
	Stilbenoid, diarylheptanoid and gingerol biosynthesis	ko00945	7
	Biosynthesis of amino acids	ko01230	7
	Protein processing in endoplasmic reticulum	ko04141	7
CK-D VS IBA-4	Plant hormone signal transduction	ko04075	50
	Plant‒pathogen interaction	ko04626	44
	MAPK signaling pathway - plant	ko04016	28
	Phenylpropanoid biosynthesis	ko00940	19
	Starch and sucrose metabolism	ko00500	18
	Protein processing in endoplasmic reticulum	ko04141	11
	Carbon metabolism	ko01200	8
	Valine, leucine and isoleucine degradation	ko00280	7
	Circadian rhythm - plant	ko04712	7
	Ubiquitin mediated proteolysis	ko04120	7

### Analysis of differential expression related to hormone signaling pathways in DEGs

**Fig. 7:**
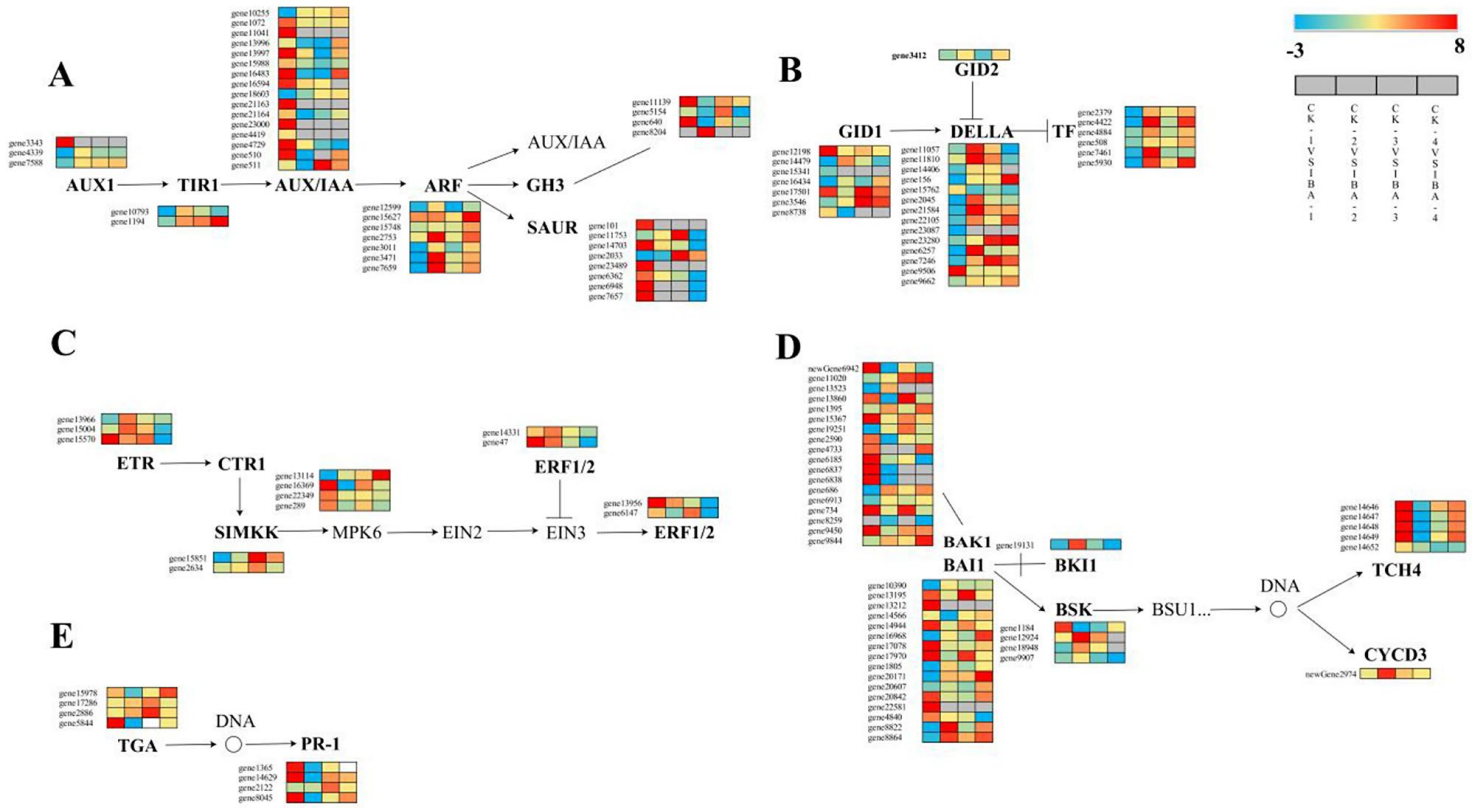
Expression of DEGs in plant hormone pathways

As indicated previously, the phytohormone signaling pathways were markedly enriched in mulberry following treatment with 1000 mg/L^− 1^ IBA. Subsequent analysis revealed that differentially expressed genes (DEGs) were predominantly enriched in auxin (Fig. 7A), gibberellin (Fig. 7B), ethylene (Fig. 7C), brassinosteroid (Fig. 7D), and salicylic acid (Fig. 7E) pathways. The findings demonstrate that these hormone signaling pathways were active in the initial stage, with the majority of genes within these pathways being upregulated during this early phase. These genes exhibited significant upregulation in the initial period, leading to the conclusion that the transition from the commencement of treatment to the tissue healing stage is critical under the influence of 1000 mg/L^− 1^ IBA. Notably, in the auxin signaling pathway, AUX/IAA gene expression was highly active, with numerous genes showing an upregulation of more than eightfold in the initial period. In contrast, the TIR1 gene did not exhibit significant upregulation until the fourth period, which may be attributed to the accumulation of TIR1 in the plant CK group. In the gibberellin signaling pathway, the pattern was distinct from the other hormone pathways; the gibberellin receptor GID1, DELLA proteins, and TF were all downregulated during the initial period, with expression levels decreasing by 1-2-fold. This suggests that DELLA proteins act as negative regulators in the gibberellin signaling pathway, thereby inhibiting plant growth and development.

### Transcription factor analysis of EDGs

**Fig. 8:**
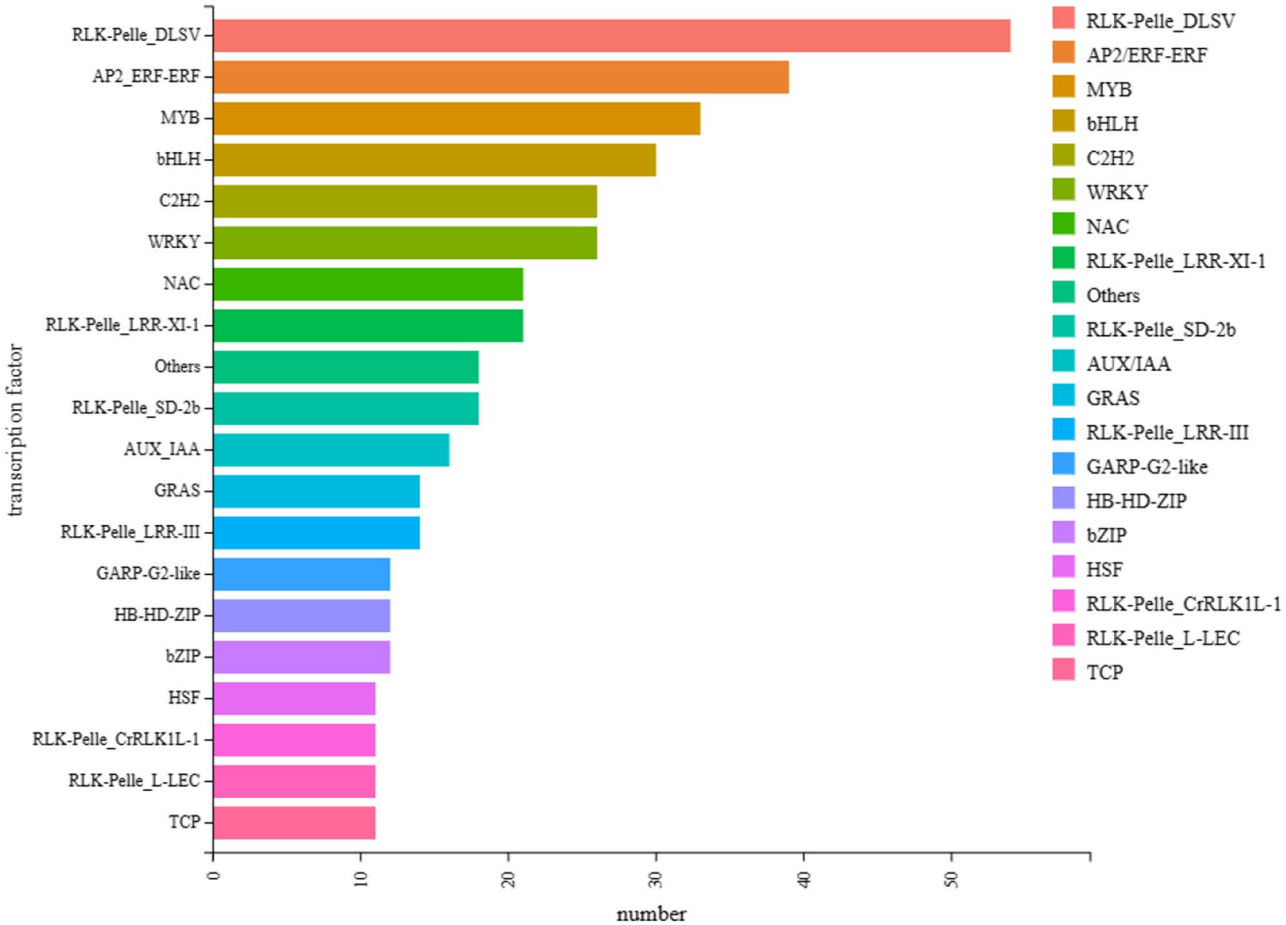
Transcription factor analysis of all DEGs in four time periods

The analysis of transcription factors among DEGs from the four periods, using Arabidopsis as the reference species, yielded a total of 410 annotated DEGs, with the top 20 by number showcased in Fig. 8. The RLK-Pelle_DLSV category contained the highest number with 54 members, followed by AP2/ERF-ERF with 39 members, and MYB with 33 members. The categories hsf, RLK-Pelle_CrRLK1L-1, and RLK-Pelle_L-LEC had the fewest members, each with only 11. It was confirmed that most of these transcription factors are closely related to plant hormones. These findings corroborate the earlier results and collectively reinforce the connection between these DEGs and plant hormones.

### Identification of key transcription factors of DEGs by WGCNA

#### Soft threshold determination and clustering of genes in gene coexpression networks

**Fig. 9:**
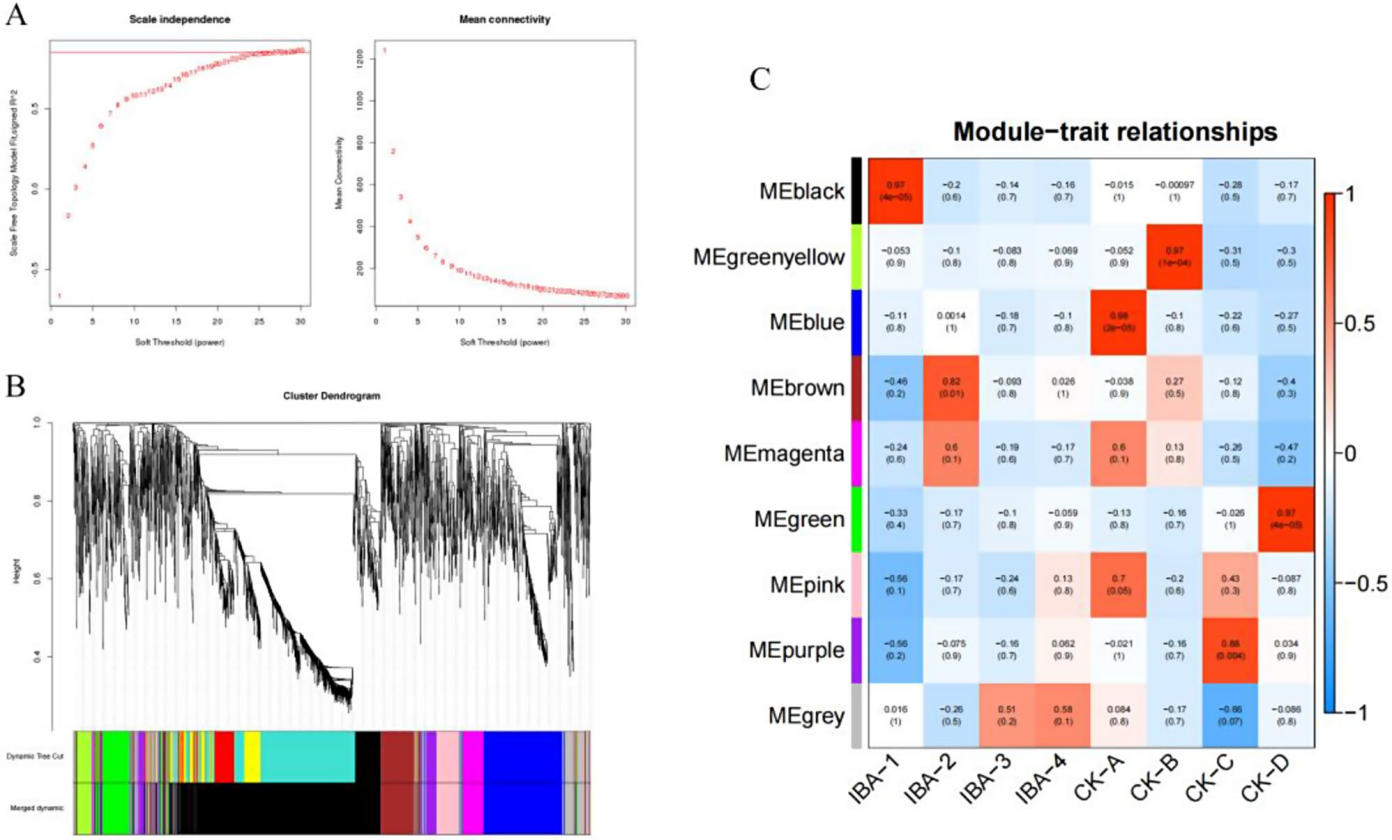
Presents a WGCNA of the gene expression matrix in mulberry. (**A**) The most appropriate soft threshold was determined by plotting scale independence and mean connectivity. (**B**) A dendrogram based on coexpression network analysis depicts the hierarchical clustering of genes, with the module colors represented on the X-axis. (**C**) The module-sample association is shown, where each row corresponds to a module color-coordinated with that in part B, and each column represents a sample. The correlations between the various modules are indicated by the values inside the colored boxes

From Fig. 9A, it is evident that a soft threshold value β of 22 resulted in a scale-free network fitting index R2 greater than 0.8, and the mean connectivity approached zero. This suggests that employing a β value of 22 enables the generation of a scale-free network that satisfies the analytical criteria; hence, β = 22 was selected for the construction of the scale-free network.

A dendrogram was generated based on the pairwise correlation of gene expression profiles (Fig. 9B). The dendrogram was truncated using dynamic tree cutting, grouping genes with similar expression patterns into the same branches, with each branch representing a distinct coexpression module. Following the amalgamation of modules with analogous expression patterns based on a threshold module similarity of 0.8, eight coexpression modules were delineated. Each module is denoted by a unique color, and the genes not classifiable into any module are represented by the color gray. The black module comprises the largest number of genes, totaling 1,081, succeeded by the blue module with 438 genes, the brown module with 192 genes, the green module with 179 genes, and the pink module with 133 genes. The magenta module includes 116 genes, the purple module comprises 105 genes, and the green-yellow module contains the smallest number of genes, with just 80 genes.

The obtained modules were analyzed for correlation with the samples, and eight modules related to different varieties and treatment times were obtained. Some of the modules were highly correlated with treatments and periods (Fig. 9C). By observing the correlation between the modules and the samples, it was found that magenta, black and brown modules were significantly positively correlated with the traits. Specifically, black and brown modules exhibited the strongest correlation, so the two modules, black and brown, were taken as the IBA treatment-related specific modules for in-depth analysis to excavate the core genes in the modules.

#### Screening and functional analysis of hub genes

**Fig. 10:**
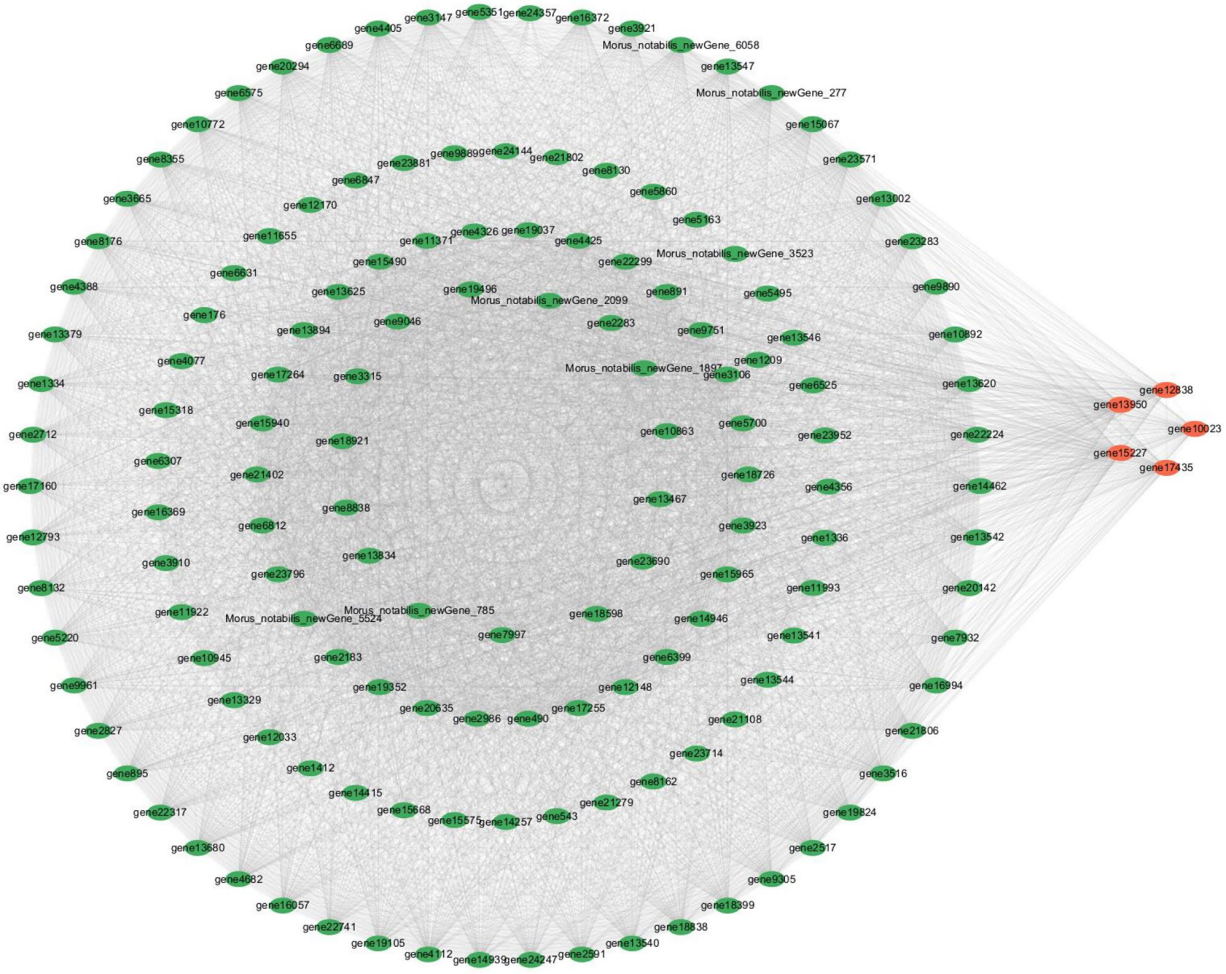
The top 150 connectivity gene networks in the black module. The orange color indicates the key transcriptional genes that have been screened

**Fig. 11:**
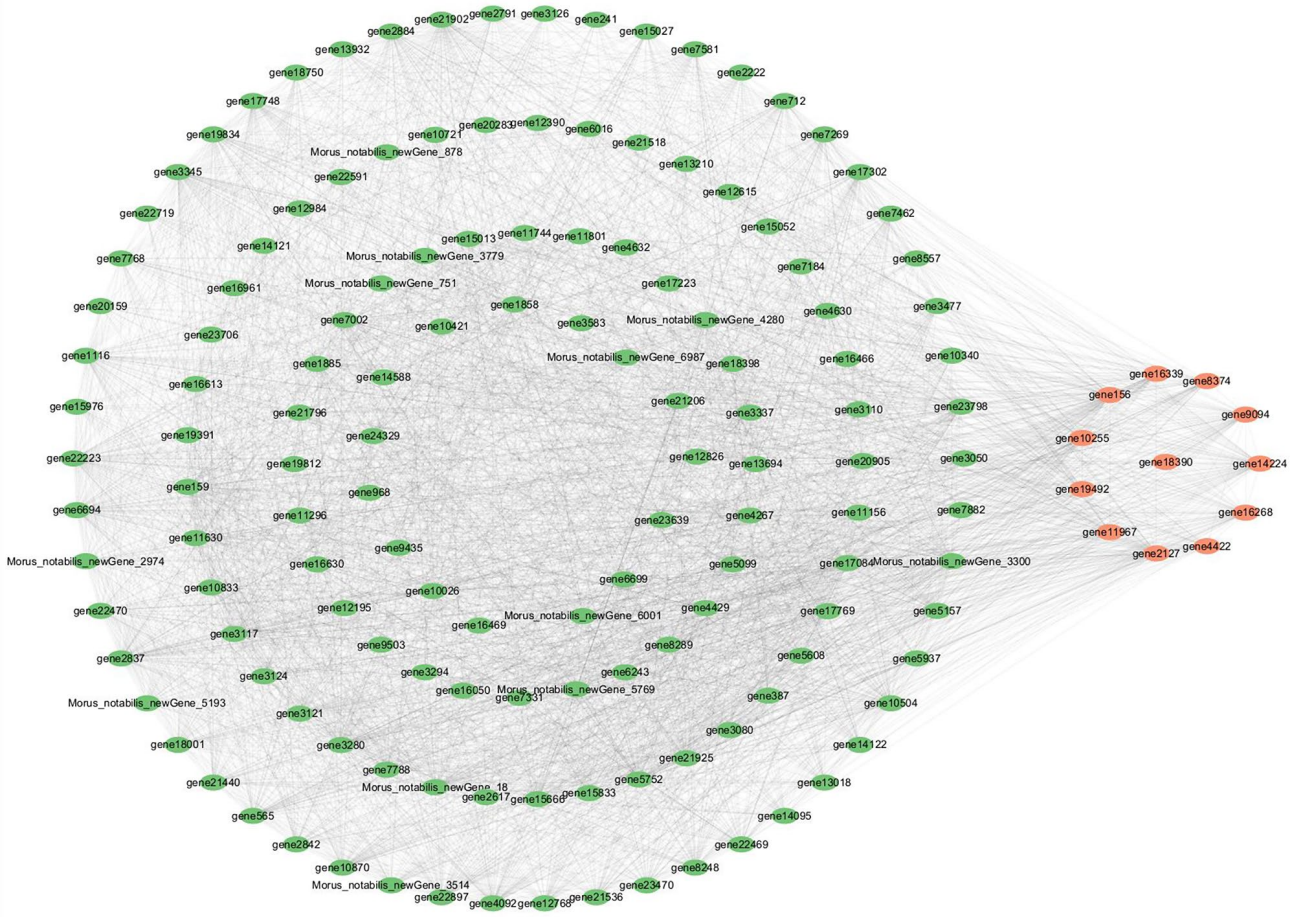
The top 150 connectivity gene networks in the brown module. The orange color indicates the key transcriptional genes that have been screened

From the top 150 most connected genes in both the black and brown modules, identified as hub genes, we screened for candidate transcription factor genes (Table 5). In the black module (Fig. 10), six transcription factor genes were identified: *WRKY53* (*gene10023*), *WRKY28* (*gene12838*), *MYB4* (g*ene13950*), *NAC7* (*gene15227*), *WRKY35* (*gene17435*), and *ERF71* (*gene18921*). In the brown module, twelve transcription factor genes were found: *IAA21* (*gene10255*), *SUVH4* (*gene11967*), *SPL14* (*gene14224*), *SHR* (*gene156*), *HAT4* (*gene16268*), *MYB2* (*gene16339*), *NAC60* (*gene18390*), *TCP4* (*gene19492*), *DOF2.4* (*gene2127*), *bHLH106* (*gene4422*), *ASIL2* (*gene8374*) and *TCP7* (*gene9094*). These 18 transcription factor genes are members of 13 distinct gene families (Fig. 11), which include three from the WRKY family (*WRKY53*, *WRKY28*, *WRKY35*); 2 NAC family (*NAC7*, *NAC60*); 2 MYB family (*MYB2*, *MYB4*); 2 TCP family (*TCP7*); 1 AP2/ERF family (*ERF71*); 1 AUX/IAA family (*IAA21*); 1 SET family (*SUVH4*); 1 bHLH family (*bHLH106*); 1 GRAS family (*SHR*); 1 SBP family (*SPL14*); 1 HB-HD-ZIP family (*HAT4*); 1 C2C2-Dof family (*DOF2.4*); and 1 Trihelix family (*ASIL2*). Upon reviewing their functions, we determined that most are intimately associated with hormone production and root development, suggesting that these transcription factor genes play a crucial role in the formation of ARs in mulberry. The specific expression of these genes alters the levels of endogenous hormones, thereby significantly enhancing the ARs formation in mulberry cuttings.

**Table 5:** Functional annotation of core transcription factors in correlation-specific modules

Mode	Gene ID	ID in Morus notabilis	Gene name	Gene function
black	*gene10023*	*LOC21394712*	*WRKY53*	*WRKY53* and *CRK5* are antagonistic regulators of chlorophyll synthesis/degradation, senescence, and stomatal conductance.
	*gene12838*	*LOC21393921*	*WRKY28*	Involved in the activation of salicylic acid biosynthesis genes ICS1 and *PBS3*. In the ovule, it is expressed in hypodermal somatic cells and appears to play a role in supression of megasporocyte cell fate. In the leaf if is upstream of *FHY3* and regulates light-mediated leaf senescence.
	*gene13950*	*LOC21407256*	*MYB4*	Encodes a R2R3 MYB protein which is involved in the response to UV-B. It functions as a repressor of target gene expression.
	*gene17435*	*LOC21404074*	*NAC7*	Involved in xylem formation by promoting the expression of secondary wall-associated transcription factors and of genes involved in secondary wall biosynthesis and programmed cell death.
	*gene15227*	*LOC21394526*	*WRKY35*	Involved in thermomorphogenesis.
	*gene18921*	*LOC21384619*	*ERF71*	The protein contains one AP2 domain. There are 5 members in this subfamily including *RAP2.2* AND *RAP2.12*. It plays a role in hypoxia-induced root slanting.
brown	*gene10255*	*LOC21408902*	*IAA21*	Activates expression of *IAA1* and *IAA9* in the presence of auxin. Mutants affect blue light and gravitropic and auxin mediated growth responses. Together with *AUX19*, it is involved in the response to ethylene.
	*gene11967*	*LOC21396328*	*SUVH4*	Ncodes a histone 3 lysine 9 specific methyltransferase involved in the maintenance of DNA methylation. *SUVH4/KYP* is a *SU(VAR)3–9* homolog, a SET domain protein.
	*gene14224*	*LOC21401011*	*SPL14*	It unctions as a transcriptional regulator that plays a role not only in sensitivity to FB1 but also in the development of normal plant architecture. The mRNA is cell-to-cell mobile.
	*gene156*	*LOC21399225*	*SHR*	Involved in radial organization of the root and shoot axial organs.
	*gene16268*	*LOC21406810*	*HAT4*	Probable transcription factor involved in the negative regulation of cell elongation and specific cell proliferation processes such as lateral root formation and secondary growth of the vascular system.
	*gene16339*	*LOC21385892*	*MYB2*	Encodes a MYB transcription factor that possesses an R2R3 MYB DNA binding domain and is known to regulate the expression of salt- and dehydration-responsive genes. Has been shown to bind calmodulin.
	*gene18390*	*LOC21402539*	*NAC60*	Represses sugar-induced *ABI5* transcription. Nonfunctional mutation of ABI5, the core transcription factor for abscisic acid signal transduction, also resulted in a phenotype of reduced root elongation and Pi content under low phosphorus conditions.
	*gene19492*	*LOC21389582*	*TCP4*	TCP4 can directly bind to the promoter of *SAUR* gene and activate its expression.
	*gene2127*	*LOC21403174*	*DOF2.4*	Regulates the development of plant branches, regulates the development of vascular bundles
	*gene4422*	*LOC21397054*	*bHLH106*	Involved in regulating the development of secondary xylem.
	*gene8374*	*LOC21406732*	*ASIL2*	Function as an inhibitor of seed ripening process
	*gene9094*	*LOC21388262*	*TCP7*	Transcription factor which plays an important role during leaf and hypocotyl development

#### Promoter analysis of ARs development related transcription factor genes

**Fig. 12:**
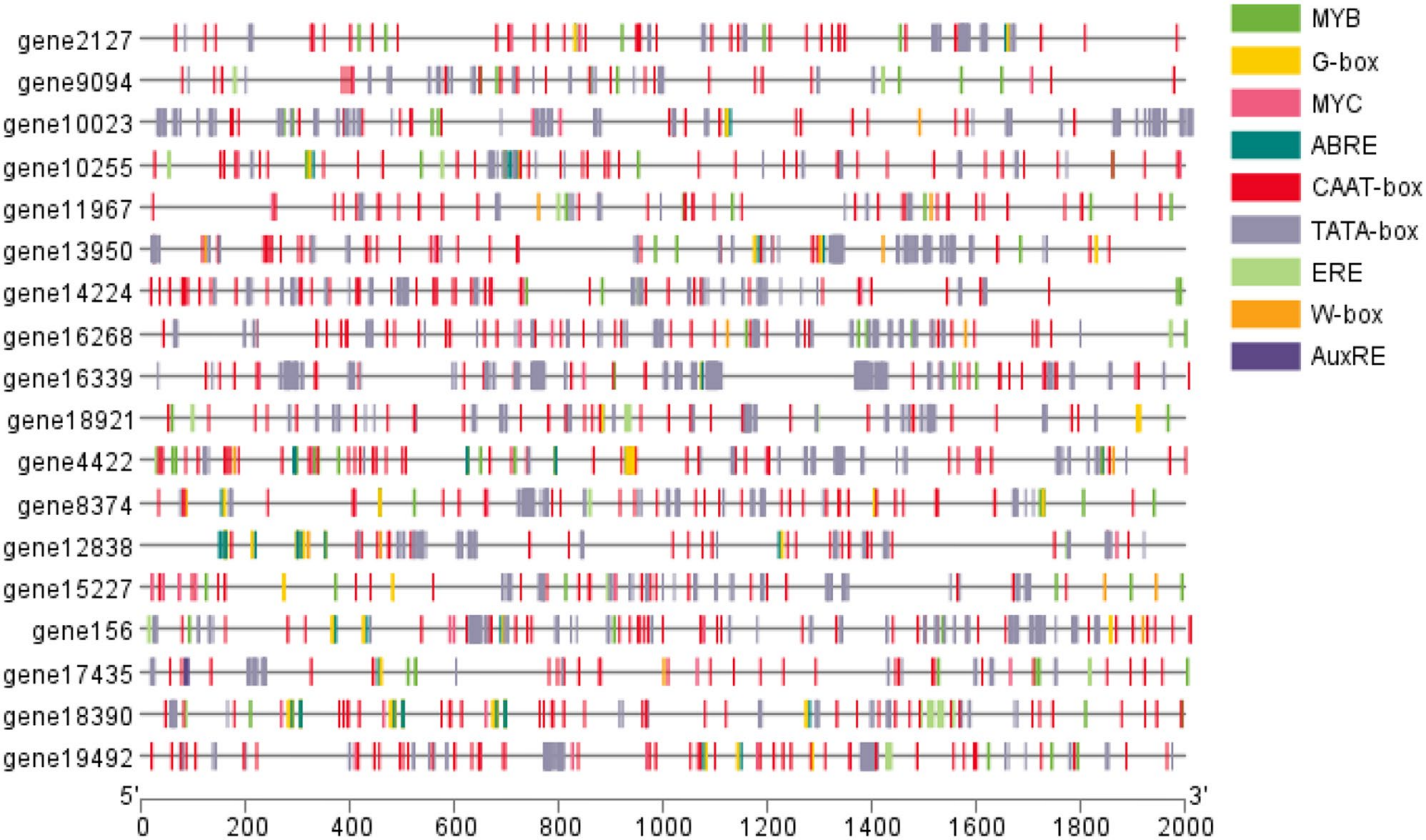
Analysis of promoter binding sites of genes associated with ARs development in co-expression networks

To further investigate the relationship between transcription factors and genes involved in plant hormone biosynthesis and signal transduction, we performed cis-acting regulatory element prediction analysis on the sequences approximately 2000 bp upstream of these genes using the PlantCARE database, the results are shown in Fig. 12. As expected, in the black and brown modules, nearly all co-expressed hormone-related genes and promoters of genes associated with ARs formation contained cis-elements such as ERE-motif, MYB-motif, G-box, W-box, MYC-motif, and ARR-motif. The repeatability of these elements suggests that these genes are not just acting individually, but are more likely to be closely related to the regulation of root growth.

### Real-time PCR validation of DEGs

**Fig. 13:**
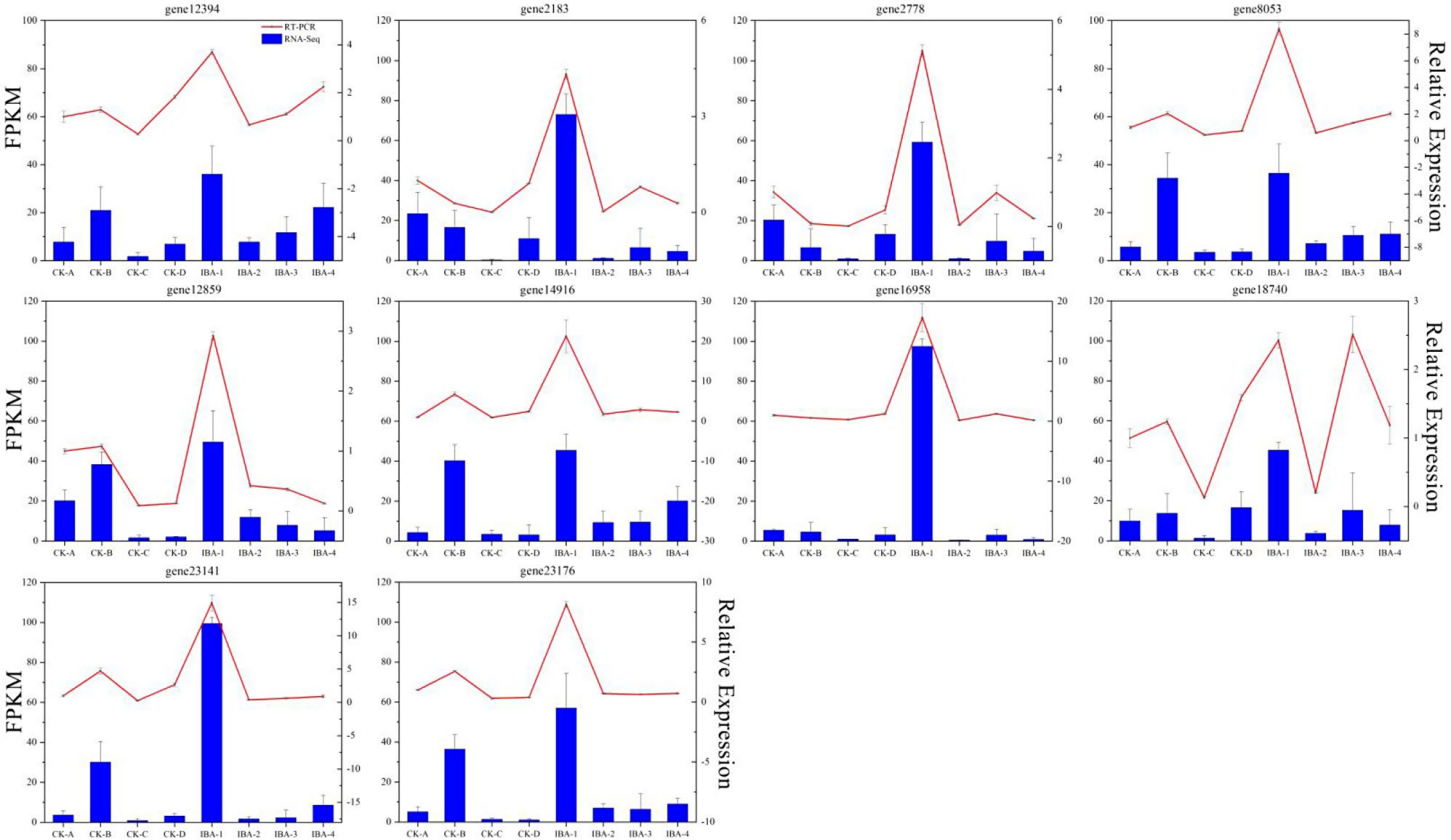
Correlation analysis between the RT‒qPCR and RNA-seq results. The curve shows the results of RT‒qPCR, and the histogram shows the results of RNA-seq. The leftmost axis represents the expression level of transcriptome sequencing, and the rightmost axis represents the expression level of RT-qPCR

To validate the RNA-seq results, 10 DEGs were randomly selected for RT-qPCR verification. The expression patterns of these genes were consistent between RNA-seq and RT-qPCR analyses, the result is shown in Fig. 13, which confirmed the accuracy and scientific validity of our experiment.

## Discussion

### The expression of endogenous hormones in plants is closely related to the formation and development of ARs

Cutting is a widely used method of vegetative propagation in horticulture. The success of this method hinges on the regenerative capacity of plant tissues. Plant hormones play an essential regulatory role by interacting with transcription factors and other regulatory elements to direct cell division, morphogenesis, and functional differentiation, ultimately leading to the regeneration of roots and shoots following callus formation [44]. In this study, a structure resembling a callus formed at the wound site of the cuttings, from which ARs subsequently developed. Previous research has indicated that endogenous hormones do not act independently in cuttings; rather, a synergistic action of multiple endogenous hormones is necessary to promote rooting and the initiation of shoots [45]. Among these, IAA is a pivotal regulator of ARs formation post-cutting, while cytokinins, jasmonic acid, gibberellins, brassinosteroids, ethylene, and other hormones are known to induce or enhance the initiation of root primordia and the formation of ARs through interactions with auxins and cytokinins [46].

In the hormone-related gene expression analysis of mulberry cuttings, differentially expressed genes were identified across the five hormonal pathways—auxin, gibberellin, ethylene, brassinosteroids, and salicylic acid. This finding suggests that these hormones participate to varying extents and through diverse mechanisms in this complex biological process. The endogenous hormone-associated genes related to IAA, gibberellin, and BR were most prevalent, with their biosynthesis and signal transduction genes upregulated across all four cutting time points. This upregulation suggests an increase in their levels and signaling activity, which may trigger cellular differentiation in mulberry leaves and the formation of ARs in cuttings. Previous research has shown that the balance between growth-promoting and growth-inhibiting hormones influences the formation of ARs [47]. In this study, the biosynthesis genes for IAA and gibberellin were found to be upregulated at day 10 post-cutting, with a greater number and a higher ratio of upregulated genes for IAA than for gibberellin, implying that a high IAA/ gibberellin ratio may be favorable for ARs development in mulberry. Additionally, BR signaling genes primarily facilitated the formation of ARs in mulberry cuttings through signal transduction. The expression patterns of the BR signaling gene *BRI1* and the gibberellin biosynthesis gene *KAO2* were consistent in the later stages of cutting, suggesting that BR may enhance gibberellin biosynthesis by upregulating *BRI1* expression. This aligns with findings that BR induces the expression of *GA20ox2* in rice seedlings by increasing the levels of the signal transduction protein BRI1, which raises the levels of bioactive gibberellin and promotes the formation of ARs [48].

###  Transcription factors WRKY, NAC, MYB and TCP are closely related to the formation and development of ARs

Studies have shown that *WRKY75*, a member of the WRKY gene family, regulates the activity of phosphatases at the transcriptional level, thereby influencing the dynamics of auxin transport and lateral root development [49]. In *Arabidopsis thaliana*, *WRKY23* has been identified as promoting localized flavonol biosynthesis, which facilitates root growth and maturation. This process is regulated by auxin through the transcriptional responses of *ARF7* and *ARF19* [50]. The wheat transcription factor TaNAC2-5 A binds directly to the promoter regions of genes encoding nitrate transporter and glutamine synthetase, enhancing root proliferation and the rate of nitrate uptake. This, in turn, improves nitrogen acquisition and ultimately increases grain yield [51]. In Arabidopsis, the transcription factor NAC1 is specifically induced by wounding in leaf explants and aids in the regeneration of the root apex [52]. In the same species, the MYB family gene *MYB77* potentially affects the number of lateral roots through its interaction with *ARF7* [53]. Furthermore, *MYB15* expression is initially upregulated in primary callus and later downregulated during rerooting, indicating its role in regulating callus-induced differentiation in tea plants [54]. Aguilar-Martinez et al. [55] reported that *AtBRC1/TCP18* interacts with both auxin and strigolactone pathways, contributing to the regulation of plant branching. In cotton, the type I protein GbTCP, analogous in function to *AtTCP15*, has been shown to reduce jasmonic acid levels and inhibit fiber elongation when silenced, while its overexpression in *Arabidopsis thaliana* promotes root hair elongation [56]. Notably, in mulberry, these transcription factors are significantly associated with auxin within the network, suggesting a primary interaction with relevant genes during the induction and elongation phases of ARs formation in tea cuttings and playing a role in the regulation of ARs regeneration. Promoter analysis supports this hypothesis, indicating a necessity for further experiments to elucidate the molecular mechanisms and physiological roles of these regulatory factors in ARs regeneration.

### Plant hormones work together to induce changes in plant roots

An exploratory analysis of the cis-regulatory elements associated with 18 transcription factors revealed that in the black and brown modules, nearly all co-expressed hormone-related genes and promoters of genes associated with ARs formation contained cis-elements such as ERE-motif, MYB-motif, G-box, W-box, MYC-motif, and ARR-motif. Typically, the W-box (TTGAC) is recognized as a cis-acting element that interacts with WRKY transcription factors. Previous binding assays have confirmed that WRKY transcription factors can bind to G-box cis-elements, playing a critical role in the signal transduction of plant hormones such asabscisic acid, salicylic acid, and jasmonic acid [57, 58]. Notably, ABRE-motifs, involved in the abscisic acid response, were identified in *gene18390* and *gene19492*, indicating a strong association with abscisic acid [59]. The AuxRE motif, essential for the auxin pathway, was found in *gene17435*, with auxin response factors selectively binding to the AuxRE motif to regulate auxin signals [60]. These findings suggest that in addition to governing downstream genes of this pathway, the endogenous hormones associated with ARs may participate in potential cross-regulation, possibly influencing the expression of genes within the plant hormone signal transduction pathway.

## Conclusion

By analyzing RNA sequencing data from mulberry phloem before and after IBA treatment, we identified differentially expressed genes. Subsequently, we discovered 18 transcription factors that potentially promote ARs formation. These transcription factors, belonging to the WRKY, NAC, MYB, and TCP transcription factor families, are closely associated with the development of ARs. Exploratory analysis of the cis-regulatory elements associated with these transcription factors suggests their potential joint regulation of plant hormone signal transduction and a ARs formation. These families may play a pivotal role in the generation of ARs, warranting further study and exploration.

## Data Availability

No datasets were generated or analysed during the current study.
